# Mutations in the non-structural protein region contribute to intra-genotypic evolution of enterovirus 71

**DOI:** 10.1186/1423-0127-21-33

**Published:** 2014-04-26

**Authors:** Sheng-Wen Huang, Hui-Li Cheng, Hsin-Yi Hsieh, Chia-Lun Chang, Huey-Pin Tsai, Pin-Hwa Kuo, Shih-Min Wang, Ching-Chuan Liu, Ih-Jen Su, Jen-Ren Wang

**Affiliations:** 1 Center of Infectious Disease and Signaling Research, National Cheng Kung University Hospital, Tainan, Taiwan; 2 Department of Medical Laboratory Science and Biotechnology, National Cheng Kung University Hospital, One University Road, Tainan 701, Taiwan; 3 Department of Emergency Medicine, National Cheng Kung University Hospital, Tainan, Taiwan; 4 Department of Pediatrics, National Cheng Kung University, Tainan, Taiwan; 5 Department of Pathology, National Cheng Kung University Hospital, Tainan, Taiwan; 6 National Institute of Infectious Diseases and Vaccinology, National Health Research Institutes, Tainan, Taiwan

**Keywords:** Enterovirus 71, Intra-genotypic evolution

## Abstract

**Background:**

Clinical manifestations of enterovirus 71 (EV71) range from herpangina, hand-foot-and-mouth disease (HFMD), to severe neurological complications. Unlike the situation of switching genotypes seen in EV71 outbreaks during 1998–2008 in Taiwan, genotype B5 was responsible for two large outbreaks in 2008 and 2012, respectively. In China, by contrast, EV71 often persists as a single genotype in the population and causes frequent outbreaks. To investigate genetic changes in viral evolution, complete EV71 genome sequences were used to analyze the intra-genotypic evolution pattern in Taiwan, China, and the Netherlands.

**Results:**

Genotype B5 was predominant in Taiwan’s 2008 outbreak and was re-emergent in 2012. EV71 strains from both outbreaks were phylogenetically segregated into two lineages containing fourteen non-synonymous substitutions predominantly in the non-structural protein coding region. In China, genotype C4 was first seen in 1998 and caused the latest large outbreak in 2008. Unlike shifting genotypes in Taiwan, genotype C4 persisted with progressive drift through time. A majority of non-synonymous mutations occurred in residues located in the non-structural coding region, showing annual increases. Interestingly, genotype B1/B2 in the Netherlands showed another stepwise evolution with dramatic EV71 activity increase in 1986. Phylogeny of the VP1 coding region in 1971–1986 exhibited similar lineage turnover with genotype C4 in China; however, phylogeny of the 3D-encoding region indicated separate lineage appearing after 1983, suggesting that the 3D-encoding region of genotype B2 was derived from an unidentified ancestor that contributed to intra-genotypic evolution in the Netherlands.

**Conclusions:**

Unlike VP1 coding sequences long used for phylogenetic study of enteroviruses due to expected host immune escape, our study emphasizes a dominant role of non-synonymous mutations in non-structural protein regions that contribute to (re-)emergent genotypes in continuous stepwise evolution. Dozens of amino acid substitutions, especially in non-structural proteins, were identified via genetic changes driven through intra-genotypic evolution worldwide. These identified substitutions appeared to increase viral fitness in the population, affording valuable insights not only for viral evolution but also for prevention, control, and vaccine against EV71 infection.

## Background

Enterovirus 71 (EV71), a positive single-stranded RNA and non-enveloped virus of the *Picornaviridae* family, generally causes mild diseases: e.g., fever, hand-foot-and-mouth disease (HFMD), herpangina. Sometimes, however, those infections are associated with severe neurological complications: aseptic meningitis, encephalitis, acute flaccid paralysis, even death [1]. EV71 has caused outbreaks around the globe since its first report as EV71 genotype A in California in 1969. According to phylogenetic analysis of the VP1 sequence, EV71 can be classified into genotypes A, B0-B5, and C1-C5 [2-4]. Studies of EV71 epidemiology show B3-B5 and C2-C5 causing Asia-Pacific epidemics since 1997 [5]. In Taiwan, EV71 caused a large 1998 outbreak with 78 fatalities [6]. Before the 1998 outbreak, an EV71 genotype B1 outbreak occurred in 1986 [1]. The predominant EV71 strains in the 1998 outbreak were genotype C2, which changed to the dominant genotype B4 from 1999 to 2002. The dominant genotype switched to C4 from 2004 to 2005, and another outbreak in 2008 was identified as genotype B5. From this epidemiological history, we noticed EV71 outbreaks recurring in Taiwan every 3–5 years, each linked with genotype change [7]. Dominant genotypes have changed from B to C and C to B several times since 1998–2012 [7]; the reason behind this circulating mode of outbreaks and the question as to whether genotypes differ in antigenicity warrant further study. Another large HFMD outbreak with neurological involvement occurred in 2008 in China [8,9]; genotype C4 is reported as the orphan genotype circulating there since 1998 [10,11]. After a decade of quiescent circulation, EV71 activity surged to cause the 2008 epidemic [8,9,11,12]. Since then, EV71 outbreaks have recurred yearly in China with high morbidity and mortality [13-20]. EV71 outbreaks have been observed not only in Malaysia [21], Singapore [22,23], Japan [24], Korea [25], Australia [3,26] but also in the Netherlands [5], where epidemiology indicated genotypes B0, B1, and B2 causing successive sporadic EV71 infections during 1963–1986. In 1986, a genotype B2 outbreak occurred and then EV71 infection showed low activity over the following ten years. In 2007, infection reoccurred, with genotype C2 predominant [27]. Among these epidemics, EV71 prevalence showed two patterns: continuous shift of genotype (in Taiwan, Japan, Malaysia, and Australia) or circulation with a sole genotype (China and Vietnam) (reviewed in [28]).

VP1 is the receptor binding and immunodominant protein of EV71. Genotyping of VP1 coding sequences has been well-established not only in modern viral taxonomy but also in phylogenetic evolution of enteroviruses [29]. Phylogenetic shifts in VP1 among genotypes might affect virus-receptor binding ability, infectivity and virulence [30-35] and viral antigenic change [7,36] to escape the host immune response.

Our prior study reported inter-genotype change among EV71 predominant strains contributing to antigenic cluster shifts within outbreaks [7], which may indicate that the observed EV71 genotype switch was driven by herd immunity. Nonetheless, as EV71 showed continuous intra-genotypic evolution in a single genotype (such as C4 circulating in China) [10], genetic diversity in the capsid protein VP1 coding region mainly contributes to synonymous versus non-synonymous mutation: i.e., not all sequence changes contribute to amino acid changes in the VP1 protein, which might change virus infectivity and/or antigenicity in the host. These findings raise another question as to why a circulating single genotype with limited capsid protein diversity becomes emergent in outbreaks after persistence in the population for years. One possibility is intra-genotypic evolution causing genetic sequence change located outside the VP1 coding region, thus augmenting viral fitness to the host. Previous investigations reported that EV71 recombination was detected in non-structural protein coding sequences of predominant strains in Taiwan (1998, 2000, and 2004) [7,37,38]; China (2008) [10,39]; Singapore (2000) [38]; and Malaysia (2000) [38]. Besides recombination, as an RNA virus, EV71 lacks a proof-reading RNA polymerase which contributes to rapid sequence evolution. Viral sequence diversity rapidly expands in a whole viral genome, including the non-structural region, and becomes a source of virus adaptability for viral fitness. Since the capsid and non-structural proteins play various roles in viral replication and host-viral interaction while viral amino acid substitutions may change protein function or activity [31,32,40-42], we dynamically analyzed sequence variations which contribute to non-synonymous mutations of all viral protein coding regions. To explore trends of EV71 intra-genotypic evolution, we examined sequences of circulating strains and those causing outbreaks, using Maximum Likelihood (ML) and molecular clock phylogeny. We characterized non-synonymous mutations of genotypes B5 in Taiwan, C4 in China, and B1/B2 in the Netherlands to identify potential viral fitness determinants in intra-genotypic evolution.

## Methods

### Virus

EV71 isolated from 2008 to 2012 from patients at National Cheng Kung University Medical Center in southern Taiwan was investigated and the preparation of virus was as previously described [43].

### RNA extraction and cDNA genome amplification

Twenty EV71 isolates from patients with diverse clinical presentations were randomly selected for sequencing analyses. Viral genomic RNA was extracted from RD cell culture with a Viral RNA purification kit II (Geneaid, Taiwan) followed by reverse transcription-PCR (RT-PCR) and complete genome sequencing as earlier described [37]. Full-length sequence was determined on both 5'- and 3'-termini by 5'RACE and 3'RACE systems (Invitrogen), as per the manufacturer’s instructions. Amplified products were cloned into pGEM-T Easy (Promega) and sequenced. Full-length cDNA RT-PCR was performed with SuperScript III reverse transcriptase (Invitrogen) for reverse transcription and KOD + (Clontech) for PCR. PCR products were cloned by TOPO XL PCR kit (Invitrogen) and sequenced. Multiple sequence alignments were performed, using Clustal X v1.83.

### Phylogenetic analyses

Using the model test program in MEGA 5.2, we chose the models with the lowest BIC scores (Bayesian Information Criterion) which are considered to best describe the substitution pattern. Transition/transversion ratios were calculated as 10.43 and 7.98 for VP1 and 3D gene analysis, respectively. Phylogenetic trees according to VP1 and 3D sequences were estimated by the General Time Reversible (GTR) model of PAUP* 4.0b as previously described [44]. Statistical robustness of 1,000 data sets were analyzed, and significance of branch length was estimated by maximum-likelihood. Bayesian MCMC analysis was performed by using relaxed molecular clock (uncorrelated lognormal-distributed) and Hasegawa-Kishino-Yano (HKY) nucleotide substitution models (with BEAST software v1.8.0). Each Bayesian MCMC analysis was performed for 10,000,000 states, sampled every 10,000 states. Posterior probability was calculated with a burn-in of 1,000,000 states and a timescale was added to phylogeny history of strains to estimate dates of common ancestors.

### Nucleotide sequence accession numbers

Twenty sequences from clinical isolates in 2008–2012 in Taiwan have been deposited in GenBank sequence database and the accession numbers are KF974779-KF974798 (Additional file 1: Table S1).

## Results

### Re-emergence of genotype B5 in Taiwan

**Figure 1:**
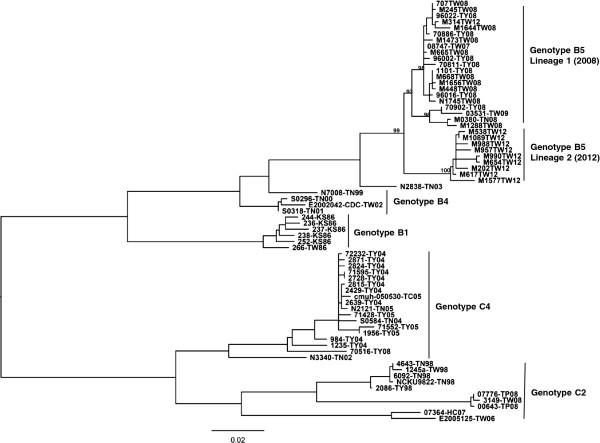
**Maximum Likelihood phylogeny of EV71 strains according to VP1 coding region in Taiwan.** Complete VP1 sequences of various genotypes in Taiwan were used to construct a phylogenetic tree as indicated. The tree is shown in decreasing order, and bootstrap values of nodes are indicated at the nodes.

**Figure 2:**
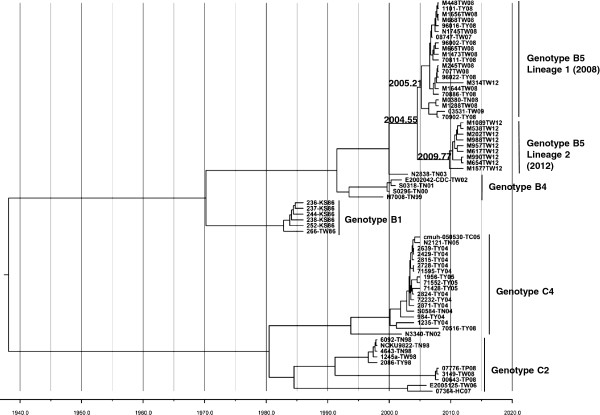
**Bayesian MCMC analysis phylogeny of EV71 strains according to VP1 coding region in Taiwan.** Complete VP1 sequences of various genotypes in Taiwan with known sampling dates were used to construct a phylogenetic tree as indicated. The tree is shown in decreasing order, and the estimated dates of common acenstors of nodes are indicated at the nodes.

Taiwan CDC enterovirus surveillance showed a large EV71 outbreak recurring in 2012, following the previous outbreak in 2008 [45]. Phylogenetic sequences of VP1 coding from 2008 and 2012 isolates indicated both epidemics were caused by genotype B5 (Figure 1). Our previous investigation reported continuous genotypic change responsible for each new outbreak in Taiwan every 2–5 years from 1998 to 2008; the genotype B5 outbreak showed a unique pattern in Taiwan’s epidemiological history, in that the same genotype precipitated large outbreaks in 2008 and 2012. To detail evolutionary trends of circulating EV71, we sequenced whole genomes of 20 isolates in both outbreaks for phylogenetic analysis. Phylogenic ML and molecular clock phylogeny targeting structural protein VP1 and non-structural protein 3D were performed to examine EV71 diversity through time. ML analysis of VP1 coding sequences (Figure 1) displayed genotype B5 isolates from 2012 as segregated into a distinct sub-lineage of genotype B5 distant from 2008 and 2009 isolates, with one exception, namely that the M314-TW12 isolate was genetically close to 2008 isolates. Non-structural 3D coding sequences displayed similar ML phylogeny with structural VP1 protein coding sequences (Additional file 2: Figure S1). To assess evolutionary change of EV71 through time, we performed Bayesian evolutionary analysis and estimated the dates of origin of both lineages in genotype B5 with an exponential growth model. The results indicated a common ancestor of B5 dated to 1999, whereas the first Taiwan isolate was detected in 2003 (Figure 2). The date of the common ancestor of the two sub-lineages in the 2008 and 2012 outbreaks was estimated to be 2004 (Figure 2). According to the date of the common ancestor of the 2012 isolates, genotype B5 continued evolving after the 2008 outbreak and developed a new sub-lineage around 2009, followed by re-emergence in 2012. Sequences of the 3D coding region showed a similar origin estimate, suggesting that the ancestor of the new sub-lineage of 2012 appeared around 2010 after the 2008 outbreak (Additional file 3: Figure S2).

To ascertain whether new sub-lineage contributes to non-synonymous substitutions, amino acid sequences of polyprotein were aligned for comparison. The capsid protein coding region showed only four sporadic amino acid substitutions: VP2_89_, VP2_177_, VP1_98_, and VP1_145_ (Table 1). Variants showed continuing evolution in the structural protein region, but no marked evolutionary pattern emerged between the 2008 and 2012 outbreaks. In contrast to four substitutions in the capsid protein coding region, the non-structural protein coding region showed fourteen amino acid substitutions: two in 2A_52_ and 2A_102_, two in 2C_243_ and 2C_257_, three in 3C_60_, 3C_96_, and 3C_182_, and seven in 3D_22_, 3D_126_, 3D_143_, 3D_228_, 3D_251_, 3D_383_ and 3D_396_ (Table 1). In addition, all these substitutions displayed obvious differential signatures between the 2008 and 2012 strains, indicating re-emergent genotype B5 in 2012 belongs to a new sub-lineage of B5 characterized by dozens of non-synonymous mutations accumulating in non-structural proteins.

**Table 1:** Amino acid sequence comparison of enterovirus 71 genotype B5 in Taiwan

**Gene**	**VP2**		**VP1**		**2A**		**2C**		**3C**			**3D**						
**Amino acid position**	**89**	**177**	**98**	**145**	**52**	**102**	**243**	**257**	**60**	**96**	**182**	**22**	**126**	**143**	**228**	**251**	**383**	**396**
*Strain No.*																		
2008																		
M314-TW08	T	I	E	G	I	A	F	D	V	A	M	R	K	F	S	V	H	K
M448-TW08	T	I	E	E	I	A	F	D	V	A	M	R	K	F	S	V	H	R
M665-TW08	T	I	E	E	I	A	F	D	V	A	M	R	K	F	S	V	H	R
M707-TW08	T	I	E	E	I	A	F	D	V	A	M	R	K	F	S	V	H	K
M1473-TW08	T	I	E	G	I	A	F	D	V	A	M	R	K	F	S	V	H	R
M380-TW08	I	M	E	G	T	A	Y	D	I	G	M	R	K	F	S	V	H	R
M245-TW08	T	I	E	E	I	A	F	D	V	A	M	R	K	F	S	V	H	K
M668-TW08	T	I	E	E	I	A	F	D	V	A	M	R	K	F	S	V	H	R
M1288-TW08	I	M	K	E	T	A	Y	D	I	G	M	R	K	F	S	V	H	R
M1644-TW08	T	I	E	E	I	A	F	D	V	A	M	R	K	S	S	V	H	K
M1656-TW08	T	I	E	E	I	A	F	D	V	A	M	R	K	F	S	V	H	R
N1745-TW08	T	I	K	E	I	A	F	D	V	A	M	R	K	F	S	V	H	R
2012																		
M538-TW12	T	I	E	Q	T	V	Y	E	I	G	T	H	R	L	A	I	Y	R
M617-TW12	T	I	E	E	T	V	Y	E	I	G	T	H	R	L	A	I	Y	R
M988-TW12	T	I	E	E	T	V	Y	E	I	G	T	H	R	L	A	I	Y	R
M990-TW12	T	I	E	Q	T	V	Y	E	I	G	T	H	R	L	A	I	H	R
M202-TW12	T	I	K	E	T	V	Y	E	I	G	T	H	R	L	A	I	Y	R
M654-TW12	T	I	E	E	T	V	Y	E	I	G	T	H	R	L	A	I	H	R
M957-TW12	T	I	E	E	T	V	Y	E	I	G	T	H	R	L	A	I	Y	K
M1089-TW12	T	I	E	E	T	V	Y	E	I	G	T	H	R	L	A	I	Y	R
M1577-TW12	T	I	E	E	T	V	Y	E	I	G	T	H	R	L	A	I	Y	R

EV71 is widely known to gain foreign gene fragments by both inter- and intra-serotypic recombination. We screened for potential viral recombination between the 2012 isolates and other enteroviruses but no obvious recombination emergent events were detected by the Recombination Detection Program (data not shown). These results suggest that sequence variants in non-structural protein regions likely arise from continuous accumulation of mutations.

### Continuing evolution of genotype C4 in China outbreaks

**Figure 3:**
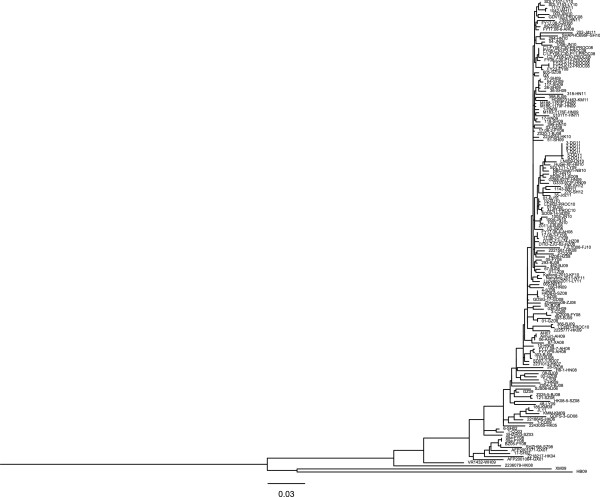
**Maximum Likelihood phylogeny of EV71 strains according to VP1 coding region in China.** A total of 154 complete VP1 sequences of genotype C4 in China were used to construct a phylogenetic tree as indicated. The tree is shown in decreasing order, and bootstrap values of nodes are indicated at the nodes.

**Figure 4:**
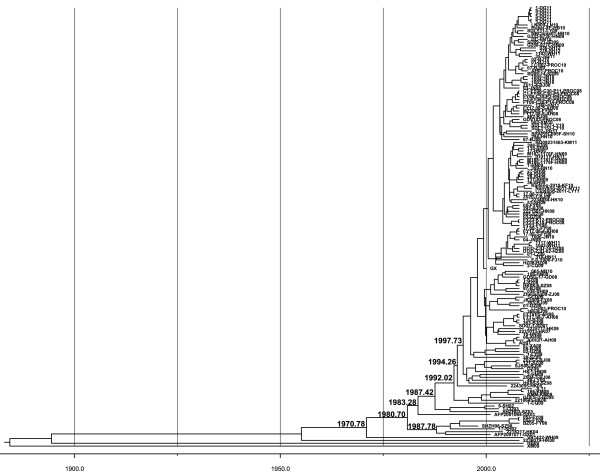
**Bayesian MCMC analysis phylogeny of EV71 strains according to VP1 coding region in China.** A total of 154 complete VP1 sequences of genotype C4 in China with known sampling dates were used to construct a phylogenetic tree as indicated. The tree is shown in decreasing order, and the estimated dates of common acenstors of nodes are indicated at the nodes.

EV71 genotype B5 accumulated evolutionary amino acid substitutions especially in non-structural proteins, causing re-emergence in the 2012 outbreak after the 2008 HFMD outbreak in Taiwan. In Mainland China since 1998, EV71 was identified in the following ten years circulating with low activity [9,46]. The latest large HFMD outbreak (in 2008) caused approximately 490,000 infections with 126 fatalities. Since then EV71 has caused annual outbreaks in China [12,19,20,47]. To examine whether similar continual turnover of non-structural proteins occured in genotype C evolution, we characterized genotype C4 evolution in Mainland China where repetitive EV71 outbreaks have been initiated by a single genotype. To compare the genetic evolution in the structural protein coding region versus that of the non-structural protein coding region, we analyzed 154 available complete sequences of Chinese EV71 strains retrieved from the GenBank database. ML and Bayesian MCMC evolutionary analyses evaluated C4 sequence evolution in the VP1 and 3D protein coding regions. Unlike two diverse lineages of genotype B5 in the 2008 and 2012 outbreaks in Taiwan, ML phylogenic trees of VP1 and 3D of genotype C4 from China appeared similar to ladder-like structures with progressive drifts across time (Figure 3 and Additional file 4: Figure S3). In addition, Bayesian evolutionary analysis and estimated date of common ancestor indicated genotype C4 in mainland China appearing about 1980 (Figure 4 and Additional file 5: Figure S4). The estimated date of origin indicated the common ancestor appeared 6–13 years ago, after which the virus lineage showed continual turnover year by year and accumulated mutations, which became the predominant strain in the 2008 outbreak in China.

**Figure 5:**
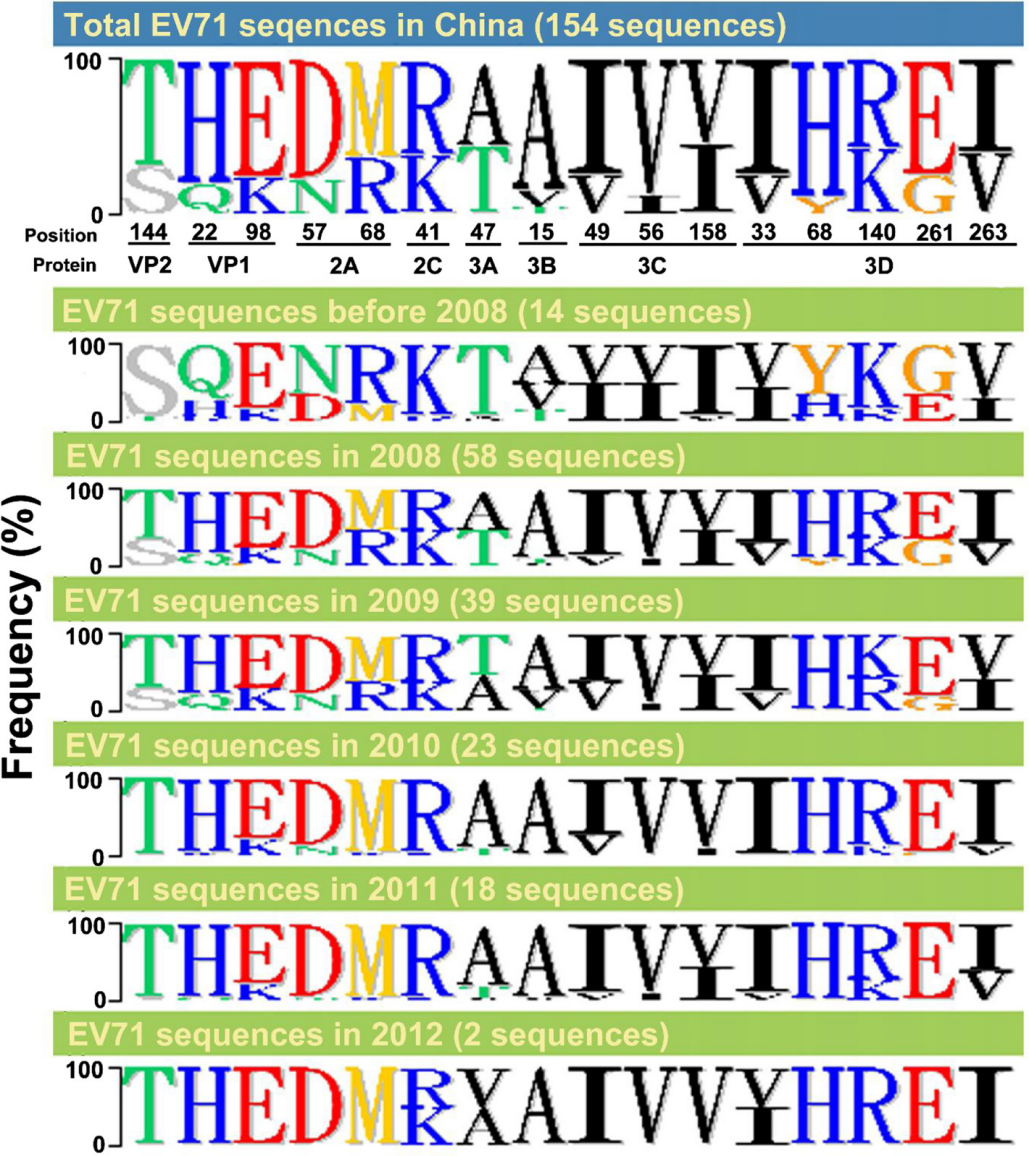
**The frequency of amino acid substitutions in polyprotein of China strains from 1998 to 2012.** Amino acid sequences were aligned by Clustal X program and the gene signature was displayed using the Phylo-mLogo program. The frequency of amino acid sequences relative to the total number of sequences in each indicated period are shown.

To analyze accumulated mutations in the evolution of the sole genotype circulating in China, we compared the viral polyprotein amino acid sequences occuring through time until 2012. A total of 16 residues with amino acid changes after the 2008 outbreak were identified (Figure 5): S to T in VP2_144_, Q to H in VP1_22_, K to E in VP1_98_, N to D in 2A_57_, R to M in 2A_68_, K to M in 2C_41_, T to A in 3A_47_, V to A in 3B_15_, V to I in 3C_49_, I to V in 3C_56_, I to V in 3C_158_, V to I in 3D_33_, Y to H in 3D_68_, K to R in 3D_140_, G to E in 3D_261_, and V to I in 3D_263_. Instead of any obvious dominant sequence change between Taiwan’s outbreaks in 2008 and 2012 as mentioned, these residues were gradually replaced by new amino acids each year; most became dominant sequences in 2011 or 2012, correlating with continual lineage turnover in ML phylogeny (Figure 3 and Additional file 4: Figure S3). Notably, most amino acid substitutions occurred in the coding regions of non-structural proteins rather than those of structural proteins, indicating EV71 accumulated mainly non-structural protein substitutions in the process of intra-genotypic evolution.

### Intra-genotype B evolution in Netherlands

**Figure 6:**
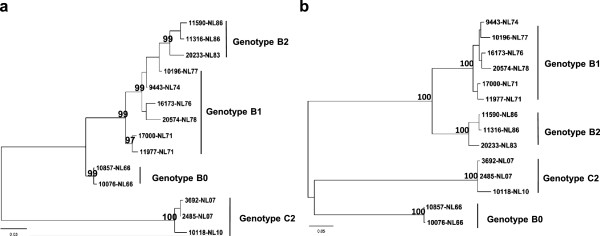
**Maximum Likelihood phylogeny of EV71 strains according to VP1 and 3D coding region in the Netherlands.** Complete VP1 **(a)** and 3D **(b)** sequences of genotype B1/B2 from the Netherlands were used to construct phylogenetic trees as indicated. The trees are shown in decreasing order, and bootstrap values of nodes are indicated at the nodes.

**Figure 7:**
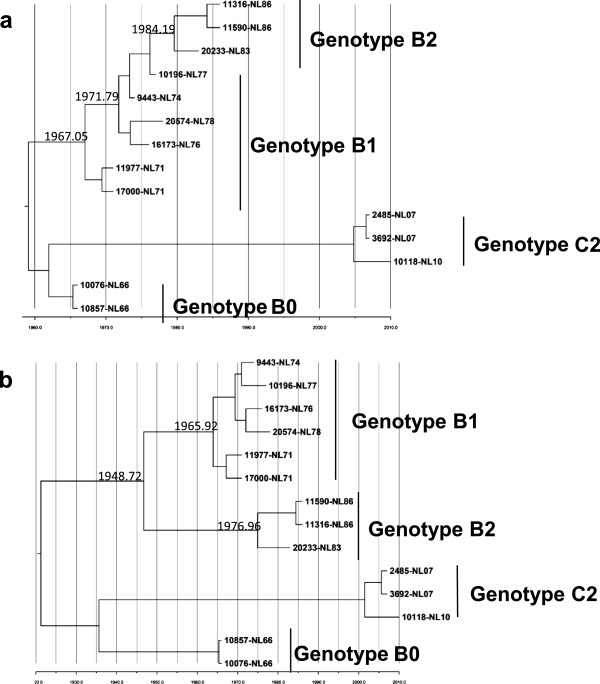
**Bayesian MCMC analysis phylogeny of EV71 strains according to VP1 and 3D coding region in the Netherlands.** Complete VP1 **(a)** and 3D **(b)** sequences of genotype B1/B2 in the Netherlands with known sampling dates were used to construct a phylogenetic tree with time line as indicated. The trees are shown in decreasing order, and the estimated dates of common acenstors of nodes are indicated at the nodes.

Similar continuous lineage turnover surfaced in the Netherlands, where EV71 changed among genotype B0, B1, and B2 in 1963–1986, with B2 as the predominant strain in the 1986 outbreak [4,5]. To examine the evolutionary pattern in EV71 of genotype B and to compare with those observed in genotype B5 in Taiwan and genotype C4 in China, we retrieved 14 complete sequences from the Netherlands published in Genbank, comparing their VP1 and 3D coding regions by ML phylogenic and Bayesian evolutionary analysis. VP1 coding region sequences revealed three major clades, B0, B1/B2 and C2, in the ML phylogeny tree (Figure 6a). The B1/B2 clade in ML phylogeny showed ladder-like evolution similar to C4 in China; viruses continuously evolved along the phylogenetic trunk. The common ancestor of B1/B2 was estimated to date around 1971 (Figure 7a). However, ML phylogeny of 3D sequences displayed a diverse phylogenetic tree: B1 and B2 did not evolve a single trunk but divided into two branches (Figure 6b). Rather than sharing one common ancestor among VP1 sequences of genotype B1/B2, 3D sequences of B2 strains causing the 1986 outbreak in the Netherlands have a distinct ancestor dated in 1976 (Figure 7b), suggesting that genotype B2 might have acquired 3D genome sequences from an ancestor other than B1. To determine whether diverse nucleotide sequences contribute to amino acid substitutions, amino acid sequences of B1/B2 were aligned for comparison. A total of six successive substitutions in VP4 and VP1 were found in the structural region through time (Table 2). The non-structural region contained 23 residue changes in amino acid sequences. Residues, 3D_45_, 3D_93_, 3D_105_, 3D_251_, 3D_312_, and 3D_346_ contained unique sequence signatures in the predominant strains of the Netherlands’ 1986 outbreak, in contrast to those before 1978 in the Netherlands. Therefore, with 3D phylogeny displaying a diverse branch of genotype B2, the results suggest these amino acid residues may be contributed by another ancestor’s genome, along with changing viral fitness of B1 strain to cause the EV71 outbreak in 1986.

**Table 2:** Amino acid sequence comparison of enterovirus 71 genotype B1/B2 in Netherlands

**Gene**	**VP4**	**VP1**					**2A**				**2B**	**2C**							**3A**		**3C**	**3D**							
**Amino acid position**	**60**	**16**	**145**	**164**	**249**	**282**	**25**	**57**	**60**	**102**	**60**	**13**	**44**	**48**	**103**	**146**	**311**	**316**	**11**	**60**	**8**	**45**	**93**	**105**	**134**	**143**	**251**	**312**	**346**
*Strain no.*																													
17000-NL71	I	V	E	D	I	N	R	N	T	V	E	N	V	N	A	V	V	N	S	V	S	N	N	E	L	L	**T**	**R**	**R**
11977-NL71	I	V	Q	D	I	D	R	D	T	V	E	N	V	N	A	V	V	N	S	V	S	N	N	E	S	L	**T**	**R**	**R**
9443-NL74	V	M	E	E	I	N	H	D	T	V	D	N	A	N	V	I	I	S	S	V	P	N	N	E	L	L	**T**	**R**	**R**
16173-NL76	I	M	Q	E	I	D	H	N	T	V	D	N	A	N	V	I	V	S	N	V	S	N	N	E	L	F	**T**	**R**	**R**
10196-NL77	V	V	E	E	I	N	H	D	A	A	D	N	A	N	V	I	I	S	S	A	S	X	N	E	L	F	**T**	**R**	**R**
20574-NL78	I	M	E	E	I	N	H	N	T	V	D	N	A	N	V	I	V	S	N	V	S	N	N	E	L	F	**T**	**R**	**R**
20233-NL83	I	V	E	E	V	N	H	D	T	A	D	S	A	T	V	I	I	S	S	A	P	S	K	D	A	S	**V**	**K**	**K**
11590-NL86	I	V	G	E	V	N	H	D	A	A	D	S	A	T	V	I	I	S	S	A	S	S	K	D	A	S	**V**	**K**	**K**
11316-NL86	I	V	Q	E	V	N	H	D	A	A	D	S	A	T	V	I	I	S	S	A	S	S	K	D	A	S	**V**	**K**	**K**

## Discussion

Since 1997, EV71 has caused large outbreaks in the Asia-Pacific region. According to prevalance and genetic analysis of EV71 outbreaks worldwide, the deduced evolutionary pattern included mutiple genotype shifts (reviewed in [28]) or a single genotype ciruculation [10,48]. Our prior antigenic study provides a possible explanation for re-emergence: that genotype shifts accompany antigenic changes to escape herd immunity [7]. Nonetheless, it remains unclear as to why a sole genotype can persist for a long period and then cause large outbreaks. Genbank database collected around 300 complete EV71 genome sequences during 1970–2012, allowing dynamic and global examination of viral evolution. Instead of pooling all genotype sequences from various countries avaiable in the GenBank database, we focused on strains isolated from periods and areas with EV71 (re-)emergence in single genotype, including 2008–2012 in Taiwan, 2008–2012 in China, and 1971–1986 in the Netherlands. The results affirm the gradual accumulation of mutations in genotypes B5, C4, and B1/B2 of EV71 which accompany continual lineage turnover. Virus sequences, not only in the structural but also dominant in the non-structural protein coding region, showed successive accumulation of non-synonymous mutations year by year, suggesting viral fitness increase through time subsequently leading to an outbreak. Our study also emphasizes the importance of examining the non-structural protein coding region for full understanding of EV71 evolution.

A previous study used VP1 sequences available in the GenBank database, to reconstruct the spatiotemporal epidemic history of EV71, indicating predominant strains in outbreaks circulating among the human population for 1–5 years before onset [44]. This scenario was observed in not only our Bayesian MCMC analysis but also in our epidemiology results: EV71 continuously circulated for years before large HFMD outbreaks in Taiwan, China, and the Netherlands. In addition to VP1 sequences, we analyzed 3D sequences of the same strains by Bayesian MCMC with molecular clocks to compare the evolutionary trends of VP1 and 3D sequences of genotype B5 through time. Taiwan strains indicated that the common ancestor of the predominant strains in the 2012 outbreak was estimated to date around 2009–2010. In contrast, according to sequence analysis by Bayesian MCMC, genotype C4 circulated in China for 6–13 years, then caused the 2008 outbreak. A possible reason is that viruses persistently circulate in mainland China for a long period of time, due to the large population and newborn infants becoming susceptible hosts [10]. In this time frame, EV71 appeared to evolve, increasing viral fitness in the population, leading to the 2008 outbreak in China, then becoming endemic. Sequences of B1/B2 in the Netherlands showed a distinct pattern in contrast to B5 in Taiwan and C4 in China. ML and Bayesian phylogeny according to VP1 sequences showed continual lineage replacement of circulating EV71 in the phylogenic tree until it became the predominant strain in the 1986 Netherlands outbreak. Nonetheless, 3D sequences of the same strains displayed that genotype B2 strain belonging to a terminal branch, hinting that another common ancestor in 1976 instead of genotype B1 strains, provided a genome containing the 3D coding region to genotype B2. Previous study of EV71 in the Netherlands detected no detectable recombination in the 3D coding region among genotype B2 sequences by various recoombination analyses, suggesting that some un-identified ancestor contributed the 3D coding region to B2 genome, thus improving viral fitness to the population and spawning the 1986 outbreak.

Instead of intra- or inter-genotype changes occurred in different countries (reviewed in [28]), a single genotype C4 has steadily circulated with low activity in mainland China from 1998 to 2008. Genotype C4 caused the large 2008 outbreak in China and continued causing endemics in that country. In this period, only five genotype A strains and an orphan genotype B5 strain were respectively identified in the middle and south-eastern regions of China [10,49]. As mentioned above, a large susceptible population and abundant newborns in China might be contributing factors for long time persistence of a single genotype C4. After six months of age, this cohort of newborns becomes the most susceptible population for EV71 infections while their maternal antibody begins to gradually decline. Thus, without other environmental or host pressures, the sole genotype C4 was able to persistently circulate for a long period of time in China. In contrast, smaller susceptible populations for EV71 infection in other countries leads to increases in herd immunity and genotype switch in the community. New genotypes emerge, which may exhibit increased viral fitness or diverse antigenic properties, thereby becoming the predominant strain resulting in the next wave of viral outbreak.

Sequence analysis of previous EV71 studies points to most nucleotide mutations of capsid protein coding region in the evolution as synonymous. Because of limited functional RNA secondary structure in the capsid coding region of enteroviruses [50], these synonymous mutations in the capsid coding region might not change virus property and fitness. We were therefore impelled to evaluate whether virus diversity-predisposing non-synonymous mutations were located in the non-structural instead of the structural protein region. Our sequence comparison showed that the non-structural protein coding region contained more abundant non-synonymous mutations than the structural protein coding region of B5 in Taiwan, C4 in China and B1/B2 in the Netherlands. Although the length of the non-structural protein coding region is only 1.6 times longer than that of the stuctrual protein coding region, the number of identified synonymous mutations in the non-structural region was 3.5-4.0 times those in the capsid protein region. We also estimated nucleic acid substitution rates of EV71 according to VP1 or 3D coding region sequences: the VP1 coding region showed slightly higher average substitution rates (1.661×10^-3^ ~ 3.776×10^-3^ mutations/base/year) than the 3D coding region (1.408×10^-3^ ~ 2.990×10^-3^ mutations/base/year). Therefore, intra-genotypic evolution in the non-structural protein coding region seems to show a preference in the virus genome at amino acid level. Comparing non-synonymous mutations from diverse regions indicated amino acid mutations located on residues VP1_145_, 2A_102_, 3D_143_, and 3D_251_ as identified in both genotype B5 in Taiwan and genotype B1/B2 in the Netherlands. In addition, the 2A_57_ residue was identified between genotype B1/B2 in the Netherlands and C4 in China. Residue VP1_145_ has been reported to determine receptor binding ability and mouse virulence of EV71; 2A and 3D proteins are protease and RNA-dependent RNA polymerase respectively, playing roles not only in viral translation and replication but also in antagonizing host immune response [51,52]. These mutations changed through time, suggesting improved viral adaptation to the host population. Recombination is one possible mechanism for various rapid mutations for other viruses. Several inter- and intra-serotypic EV71 recombination events have been detected in B4, C2, and C4, but our recombination analysis and earlier reports found no evidence that identified non-synonymous mutations in this study was the result of recombination between EV71 and other enteroviruses. Mutations might appear via possible selection of diverse viral reservoirs for viral fitness enhancement.

## Conclusions

Instead of analyzing partial sequences like VP1, complete genome sequencing of new EV71 strains will provide more valuable information for viral evolution and viral fitness change in enterovirus surveillance in the future. Besides examining recombination of ciruculating viruses, it is necessary to define potential amino acid substitutions in the whole viral polyprotein that determine viral fitness change. Though the mechanism of these potential fitness determinants needs further investigation, we can survey potential determinant changes to prevent and control EV71 infection. Likewise, determinants might lend insights into pathogenesis and host-virus interaction of EV71.

## Abbreviations

EV71: Enterovirus 71; HFMD: Hand foot and mouth disease; ML: Maximum likelihood methods; MCMC: Markov chain Monte Carlo (MCMC) methods.

## Competing interest

The authors declare that they have no competing interests.

## Authors’ contributions

SWH and JRW conveived, designed the experiments, and wrote the mauscript. HLC and CLC performed viral sequencing. HPT and PHK prepared virus strains. CLC, HLC, and HYH performed sequence analysis. SWH performed phylogeny analysis. SMW, CCL and IJS analyzed clinical data. All authors read and approved the final manuscript.

## Supplementary Material

Additional file 1: Table S1 EV71 reference strains obtained from GenBank database for phylogenetic analyses. Click here for file

Additional file 2: Figure S1 Maximum Likelihood phylogeny of EV71 strains according to 3D coding region in Taiwan. Complete 3D sequences of various genotypes in Taiwan were used to construct phylogenetic tree as indicated. The tree was shown in a decreasing ordering, and bootstrap values of nodes were indicated at the nodes. Click here for file

Additional file 3: Figure S2 Bayesian MCMC analysis phylogeny of EV71 strains according to 3D coding region in Taiwan. Complete 3D sequences of various genotypes in Taiwan with known sampling dates were used to construct phylogeny as indicated. The tree was shown in a decreasing ordering, and the estimated dates of common ancestors of nodes were indicated at the nodes. Click here for file

Additional file 4: Figure S3 Maximum Likelihood phylogeny of EV71 strains according to 3D coding region in China. A total of 154 complete 3D sequences of genotype C4 in China were used to construct phylogenetic tree as indicated. The tree was shown in a decreasing ordering, and bootstrap values of nodes were indicated at the nodes. Click here for file

Additional file 5: Figure S4 Bayesian MCMC analysis phylogeny of EV71 strains according to 3D coding region in China. A total of 154 complete 3D sequences of genotype C4 in China with known sampling dates were used to construct phylogeny as indicated. The tree was shown in a decreasing ordering, and the estimated dates of common ancestors of nodes were indicated at the nodes. Click here for file
