# Supplementary material for: Mutations in the non-structural protein region contribute to intra-genotypic evolution of enterovirus 71
Source: J Biomed Sci. 2014 Apr 26;21(1):33. doi: 10.1186/1423-0127-21-33 (PMC4021180; doi:10.1186/1423-0127-21-33)
Supplement: Additional file 1: Table S1 — EV71 reference strains obtained from GenBank database for phylogenetic analyses. [file 1423-0127-21-33-S1.docx]

Supplementary table. EV71 reference strains obtained from GenBank database for phylogenetic analyses

| **Strains** | **Year** | **Accession number** |
| --- | --- | --- |
| **Taiwan** |  |  |
| 6092-TN98 | 1998 | AF304459 |
| NCKU9822-TN98 | 1998 | AF136379 |
| 4643-TN98 | 1998 | AF304458 |
| 1245a-TW98 | 1998 | AF176044 |
| 2086-TY98 | 1998 | AF119796 |
| 2272-TY98 | 1998 | AF119795 |
| 70516-TY08 | 2008 | GQ231933 |
| 72232-TY04 | 2004 | GQ231940 |
| 71595-TY04 | 2004 | GQ231939 |
| 2728-TY04 | 2004 | GQ231929 |
| 2824-TY04 | 2004 | GQ231931 |
| 2815-TY04 | 2004 | GQ231930 |
| 2639-TY04 | 2004 | GQ231928 |
| 429-TY04 | 2004 | GQ231927 |
| 2871-TY04 | 2004 | GQ231932 |
| 71428-TY05 | 2005 | GQ231937 |
| 71552-TY05 | 2005 | GQ231938 |
| 1956-TY05 | 2005 | GQ231926 |
| N2121-TN05 | 2005 | FJ357374 |
| S0584-TN04 | 2004 | FJ357373 |
| E2004104-CDC-TW04 | 2004 | EF373576 |
| 984-TY04 | 2004 | DQ133458 |
| 1235-TY04 | 2004 | DQ133459 |
| N3340-TN02 | 2002 | EU131776 |
| cmuh-050530-TC05 | 2005 | HM807310 |
| 07364-HC07 | 2007 | EU527983 |
| E2005125-TW06 | 2006 | EF063152 |
| 07776-TP08 | 2008 | HM622392 |
| 00643-TP08 | 2008 | HM622391 |
| 266-KS86 | 1986 | FJ357384 |
| 252-KS86 | 1986 | FJ357383 |
| 238-KS86 | 1986 | FJ357382 |
| 244-KS86 | 1986 | FJ357381 |
| 236-KS86 | 1986 | FJ357379 |
| 237-KS86 | 1986 | FJ357380 |
| 96022-TY08 | 2008 | GQ231943 |
| 70886-TY08 | 2008 | GQ231935 |
| 08747-TW07 | 2007 | EU527985 |
| 96002-TY08 | 2008 | GQ231941 |
| 70811-TY08 | 2008 | GQ231934 |
| 96016-TY08 | 2008 | GQ231942 |
| 1101-TY08 | 2008 | GQ231925 |
| 70902-TY08 | 2008 | GQ231936 |
| 03531-TW09 | 2009 | HM622390 |
| M0380-TN08 | 2008 | FJ357385 |
| N2838-TN03 | 2003 | FJ357378 |
| N7008-TN99 | 1999 | FJ357375 |
| S0296-TN00 | 2000 | FJ357377 |
| S0318-TN01 | 2001 | FJ357376 |
| E2002042-CDC-TW02 | 2002 | EF373575 |
| M314-TW08 | 2008 | KF974779 |
| M448-TW08 | 2008 | KF974780 |
| M665-TW08 | 2008 | KF974781 |
| M707-TW08 | 2008 | KF974782 |
| M1473-TW08 | 2008 | KF974783 |
| M245-TW08 | 2008 | KF974784 |
| M668-TW08 | 2008 | KF974785 |
| M1288-TW08 | 2008 | KF974786 |
| M1644-TW08 | 2008 | KF974787 |
| M1656-TW08 | 2008 | KF974788 |
| N1745-TW08 | 2008 | KF974789 |
| M538-TW12 | 2008 | KF974790 |
| M617-TW12 | 2012 | KF974791 |
| M988-TW12 | 2012 | KF974792 |
| M990-TW12 | 2012 | KF974793 |
| M202-TW12 | 2012 | KF974794 |
| M654-TW12 | 2012 | KF974795 |
| M957-TW12 | 2012 | KF974796 |
| M1089-TW12 | 2012 | KF974797 |
| M1577-TW12 | 2012 | KF974798 |
| **China** |  |  |
| 2236079-HK08 | 2008 | KC436270 |
| VR1432-WH09 | 2009 | KC954664 |
| SHZH98-SZ98 | 1998 | AF302996 |
| 06-AH08 | 2008 | HQ611148 |
| AH07 | 2007 | KC954662 |
| Anhui1-AH09 | 2009 | GQ994988 |
| 87-XA08 | 2008 | HM003207 |
| SD07-1-SD07 | 2007 | JX678882 |
| FY17.08-7-AH08 | 2008 | JX678878 |
| FY7VP5-AH08 | 2008 | JX025561 |
| 303-BJ08 | 2008 | HM002487 |
| 110-BJ08 | 2008 | HM002486 |
| 036-SH09 | 2009 | FJ713137 |
| 10-HN08 | 2008 | GU366191 |
| 2231013-HK07 | 2007 | KC436268 |
| 97-BJ08 | 2008 | HM002485 |
| 08-BJ08 | 2008 | FJ828519 |
| 02-GZ08 | 2008 | FJ360545 |
| 1-LY09 | 2009 | JX244182 |
| 2225777-HK09 | 2009 | KC436271 |
| 276-SH12 | 2012 | KC570453 |
| 35-Jdz11 | 2011 | JQ806378 |
| 036-SH12 | 2012 | KC570452 |
| 1143-WH11 | 2011 | JX986739 |
| SD09-21-SD09 | 2009 | JX678884 |
| SD09-14-SD09 | 2009 | JX678883 |
| 07-BJ09 | 2009 | JQ319054 |
| 01-BJ10 | 2010 | JF820316 |
| LCH02-PROC10 | 2010 | JF820313 |
| 02-BJ10 | 2010 | JF820314 |
| AH01-PROC10 | 2010 | JF820315 |
| G333-972F-HN09 | 2009 | JN256060 |
| G288-927F-HN09 | 2009 | JN256059 |
| FY17.08-6-AH08 | 2008 | JX678877 |
| FY17.08-5-AH08 | 2008 | JX678876 |
| 17.08-2-FY08 | 2008 | EU703813 |
| Z011-4-BJ08 | 2008 | FJ606448 |
| FY17.08-4-AH08 | 2008 | JX678875 |
| 17.08-3-FY08 | 2008 | EU703814 |
| 17.08-1-FY08 | 2008 | EU703812 |
| DTID-ZJU-74-HZ08 | 2008 | FJ158601 |
| DTID-ZJU-62-HZ08 | 2008 | FJ158600 |
| Z020-1-BJ08 | 2008 | FJ606449 |
| 05-FY08 | 2008 | FJ439769 |
| 03-JN08 | 2008 | JF913464 |
| MZ2008-FY08 | 2008 | HQ694985 |
| FY08-C30-P14-PROC08 | 2008 | GU198371 |
| C2-FY08-C30-PROC08 | 2008 | GU198370 |
| C1-FY08-C30-P11-PROC08 | 2008 | GU198369 |
| C1-FY08-C30-P9-PROC08 | 2008 | GU198368 |
| FY08-C30-P2-PROC08 | 2008 | GU198367 |
| G398-1037F-HN09 | 2009 | JN256061 |
| HZ08-HZ08 | 2008 | HQ400942 |
| 2221581-HK08 | 2008 | KC436269 |
| 67-BJ08 | 2008 | HM002484 |
| 01-LZ09 | 2009 | GU396280 |
| 293-BJ08 | 2008 | HM053669 |
| 462-BJ09 | 2009 | HM053671 |
| 605-SZ08 | 2008 | FJ607338 |
| 17-HN09 | 2009 | JX678881 |
| 1-HN09 | 2009 | JX678880 |
| M188-1181F-HN09 | 2009 | JN256064 |
| M186-1179F-HN09 | 2009 | JN256063 |
| M183-1176F-HN09 | 2009 | JN256062 |
| 398-BJ09 | 2009 | HM002489 |
| 117-SH09 | 2009 | HQ891928 |
| 28-SH09 | 2009 | HQ891924 |
| 27-SH09 | 2009 | HQ891923 |
| 64-SH09 | 2009 | HQ891927 |
| Kaifeng-2010-KF10 | 2010 | JQ517316 |
| Nanyang-2011-NY11 | 2011 | JN052925 |
| Luoyang-2011-LY11 | 2011 | JN020147 |
| GX/LZ 08-04-GX08 | 2008 | GQ892830 |
| 04-JN08 | 2008 | HQ825317 |
| 1006-JN10 | 2010 | JQ074190 |
| 1117-WH11 | 2011 | JX986738 |
| 1042-WH11 | 2011 | JX986737 |
| SDLY11-LY09 | 2009 | JX244183 |
| LN009-LN10 | 2010 | HQ407557 |
| Hubei-XF-HB10 | 2010 | JQ804832 |
| NBChina01-NB10 | 2010 | HQ828086 |
| FY23-FY08 | 2008 | EU812515 |
| FY23-K14-PROC08 | 2008 | GU459071 |
| FY23-K12-PROC08 | 2008 | GU459070 |
| 118-SH09 | 2009 | HQ891929 |
| SDLY153-LY10 | 2010 | JX244187 |
| SDLY107-LY10 | 2010 | JX244186 |
| 003-XM10 | 2010 | HQ850973 |
| GDV103-PROC08 | 2008 | KC954663 |
| 36-SH09 | 2009 | HQ891925 |
| 2234054-HK10 | 2010 | KC436272 |
| 51-SH09 | 2009 | HQ891926 |
| 03-GZ08 | 2008 | FJ360546 |
| 9-DG11 | 2011 | JX111893 |
| 8-DG11 | 2011 | JX111892 |
| 5-DG11 | 2011 | JX111891 |
| 3-DG11 | 2011 | JX111890 |
| 2-DG11 | 2011 | JX111889 |
| 1-DG11 | 2011 | JX111888 |
| 3-CQ09 | 2009 | GQ994991 |
| JK2009-FY08 | 2008 | HQ694982 |
| GDSG-17-GD08 | 2008 | FJ194965 |
| HK08-6-SZ08 | 2008 | GQ279370 |
| 4-SZ08 | 2008 | FJ607335 |
| 1-SZ08 | 2008 | FJ607334 |
| Zhejiang08-ZJ08 | 2008 | EU864507 |
| 393-BJ09 | 2009 | HM053670 |
| 01-GZ08 | 2008 | FJ360544 |
| DC-HN10 | 2010 | HQ325852 |
| SHAPHC695F-SH10 | 2010 | JQ736684 |
| 065-NB10 | 2010 | JF830007 |
| 1005-JN10 | 2010 | JQ074189 |
| 1004-JN10 | 2010 | JQ074188 |
| 1002-JN10 | 2010 | JQ074187 |
| 2-CQ09 | 2009 | GQ994990 |
| 294-HN10 | 2010 | HM245927 |
| 106-HN09 | 2009 | HQ998852 |
| 366-BJ09 | 2009 | HM002488 |
| LCH01-PROC10 | 2010 | JF820312 |
| FJLY008-FJ10 | 2010 | HQ426649 |
| 318-HN11 | 2011 | JQ639384 |
| 399-HN10 | 2010 | HM245928 |
| 01011Y-HN11 | 2011 | JX017384 |
| HQ09231463-KM11 | 2011 | JQ316638 |
| 1360-HN11 | 2011 | JQ639383 |
| 28-SZ08 | 2008 | FJ607336 |
| 202-Jdz11 | 2011 | KC109780 |
| GDFS-3-GD08 | 2008 | FJ194964 |
| 2218645-HK06 | 2006 | KC436267 |
| 2243055-HK05 | 2005 | KC436266 |
| HK08-5-SZ08 | 2008 | GQ279369 |
| Z004-3-BJ08 | 2008 | FJ606447 |
| 48-LY09 | 2009 | JX244184 |
| H8-1-HN08 | 2008 | JQ681218 |
| 1-HN09 | 2009 | GU196833 |
| SJS06-BJ06 | 2006 | HQ129932 |
| 2-HN09 | 2009 | GQ994992 |
| 1-CQ09 | 2009 | GQ994989 |
| 6-SH02 | 2002 | JX678886 |
| 1-CQ03 | 2003 | JX678874 |
| SHZH03-SZ03 | 2003 | AY465356 |
| 17-SH02 | 2002 | JX678885 |
| AFP2001071-GX01 | 2001 | JQ742002 |
| 05a-FY08 | 2008 | HQ188292 |
| BZ05-FY08 | 2008 | HQ694983 |
| 2218217-HK04 | 2004 | KC436265 |
| AFP2001064-GX01 | 2001 | JQ742001 |
| Z025-5-BJ08 | 2008 | FJ606450 |
| 121-SZ08 | 2008 | FJ607337 |
| GZ09 | 2009 | JF799986 |
| 186-KM09 | 2009 | HQ423143 |
| JL11 | 2011 | KC414134 |
| KMM-KM09 | 2009 | HQ423142 |
| 3149-FJ08 | 2008 | JQ280307 |
| XM09 | 2009 | JN964686 |
| HB09 | 2009 | GU434678 |
| **Netherlands** |  |  |
| 365-NL00 | 2000 | AB575939 |
| 3692-NL07 | 2007 | AB575942 |
| 2485-NL07 | 2007 | AB575941 |
| 10118-NL10 | 2010 | AB575948 |
| 9612-NL91 | 1991 | AB575936 |
| 480-NL91 | 1991 | AB575935 |
| 1416-NL01 | 2001 | AB575937 |
| 10098-NL10 | 2010 | AB575938 |
| 20233-NL83 | 1983 | AB575923 |
| 11590-NL86 | 1986 | AB575928 |
| 11316-NL86 | 1986 | AB575927 |
| 16173-NL76 | 1976 | AB575916 |
| 9443-NL74 | 1974 | AB575915 |
| 20574-NL78 | 1978 | AB575918 |
| 10196-NL77 | 1977 | AB575917 |
| 17000-NL71 | 1971 | AB575914 |
| 11977-NL71 | 1971 | AB575913 |
| 10857-NL66 | 1966 | AB575912 |
| 10076-NL66 | 1966 | AB575911 |
