# Supplementary material for: Mutations in the non-structural protein region contribute to intra-genotypic evolution of enterovirus 71
Source: J Biomed Sci. 2014 Apr 26;21(1):33. doi: 10.1186/1423-0127-21-33 (PMC4021180; doi:10.1186/1423-0127-21-33)
Supplement: Additional file 3: Figure S2 — Bayesian MCMC analysis phylogeny of EV71 strains according to 3D coding region in Taiwan. Complete 3D sequences of various genotypes in Taiwan with known sampling dates were used to construct phylogeny as indicated. The tree was shown in a decreasing ordering, and the estimated dates of common ancestors of nodes were indicated at the nodes. [file 1423-0127-21-33-S3.pptx]

## Slide 1
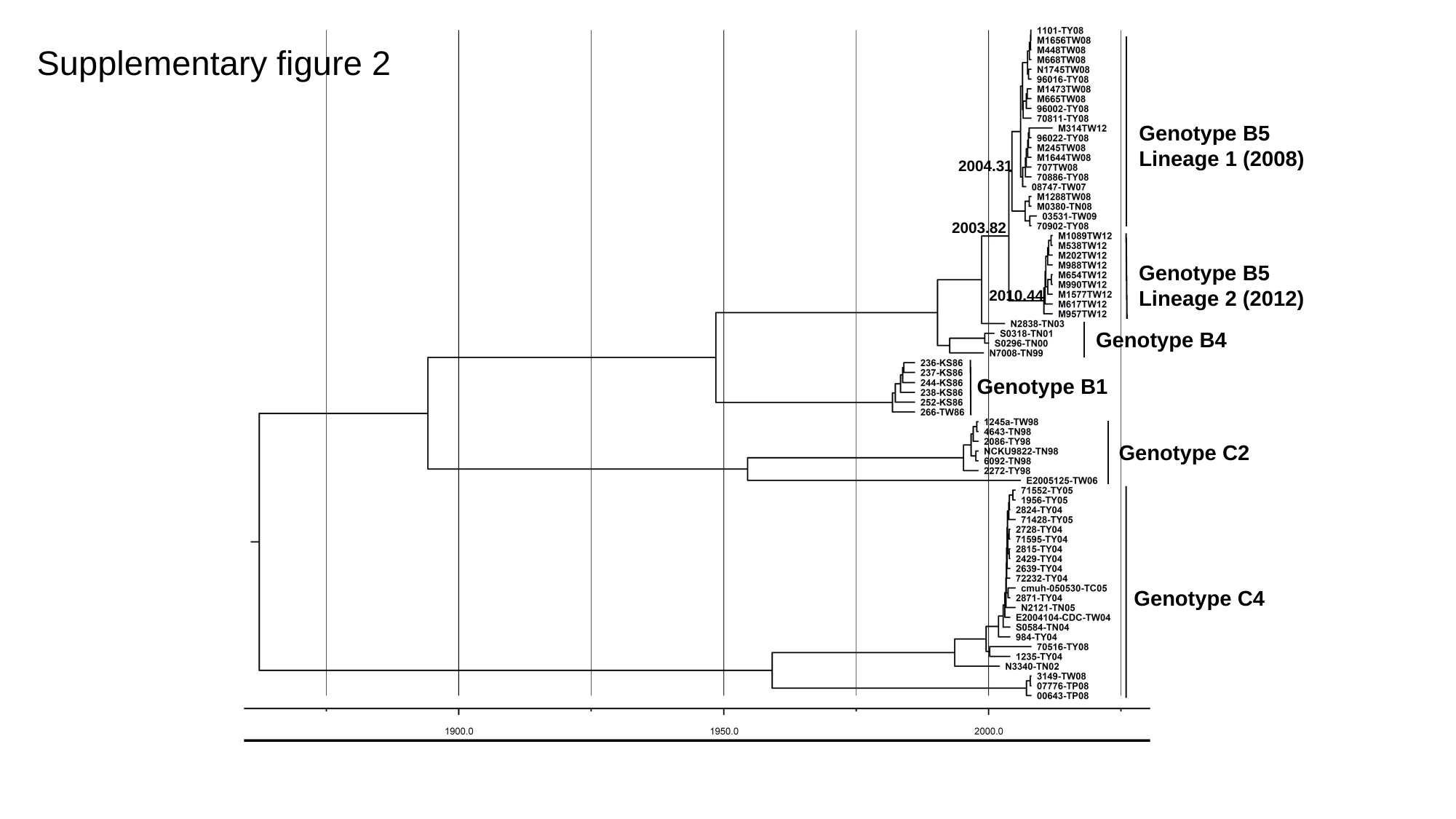

Supplementary figure 2
Genotype B5
Lineage 1 (2008)
2004.31
2003.82
Genotype B5
Lineage 2 (2012)
2010.44
Genotype B4
Genotype B1
Genotype C2
Genotype C4
