# Supplementary figures and images for: Mutations in the non-structural protein region contribute to intra-genotypic evolution of enterovirus 71
Source: J Biomed Sci. 2014 Apr 26;21(1):33. doi: 10.1186/1423-0127-21-33 (PMC4021180; doi:10.1186/1423-0127-21-33)

## Slide 1
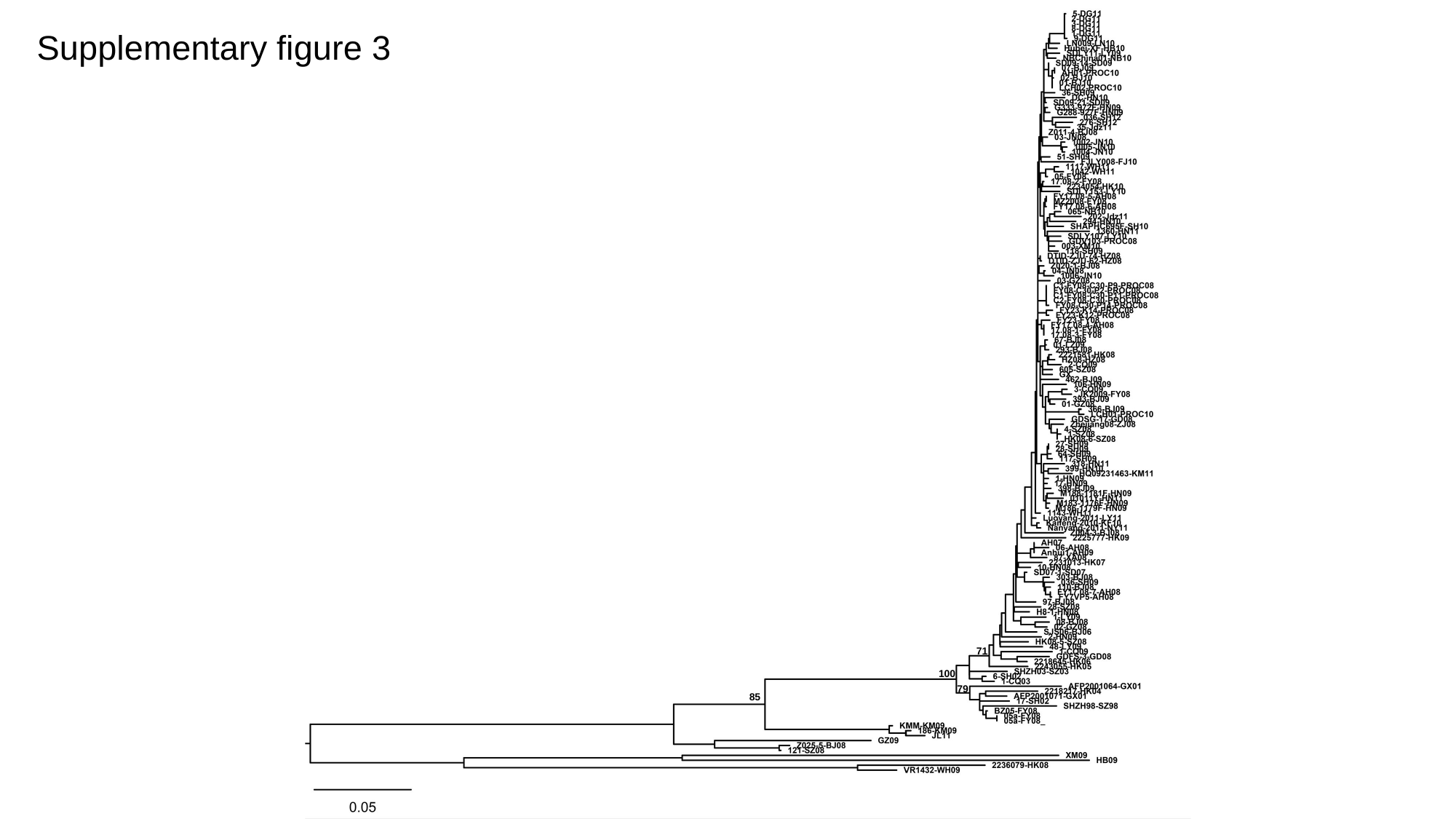

Supplementary figure 3
71
100
79
85

Supplement: Additional file 4: Figure S3 — Maximum Likelihood phylogeny of EV71 strains according to 3D coding region in China. A total of 154 complete 3D sequences of genotype C4 in China were used to construct phylogenetic tree as indicated. The tree was shown in a decreasing ordering, and bootstrap values of nodes were indicated at the nodes. [file 1423-0127-21-33-S4.pptx]

## Slide 1
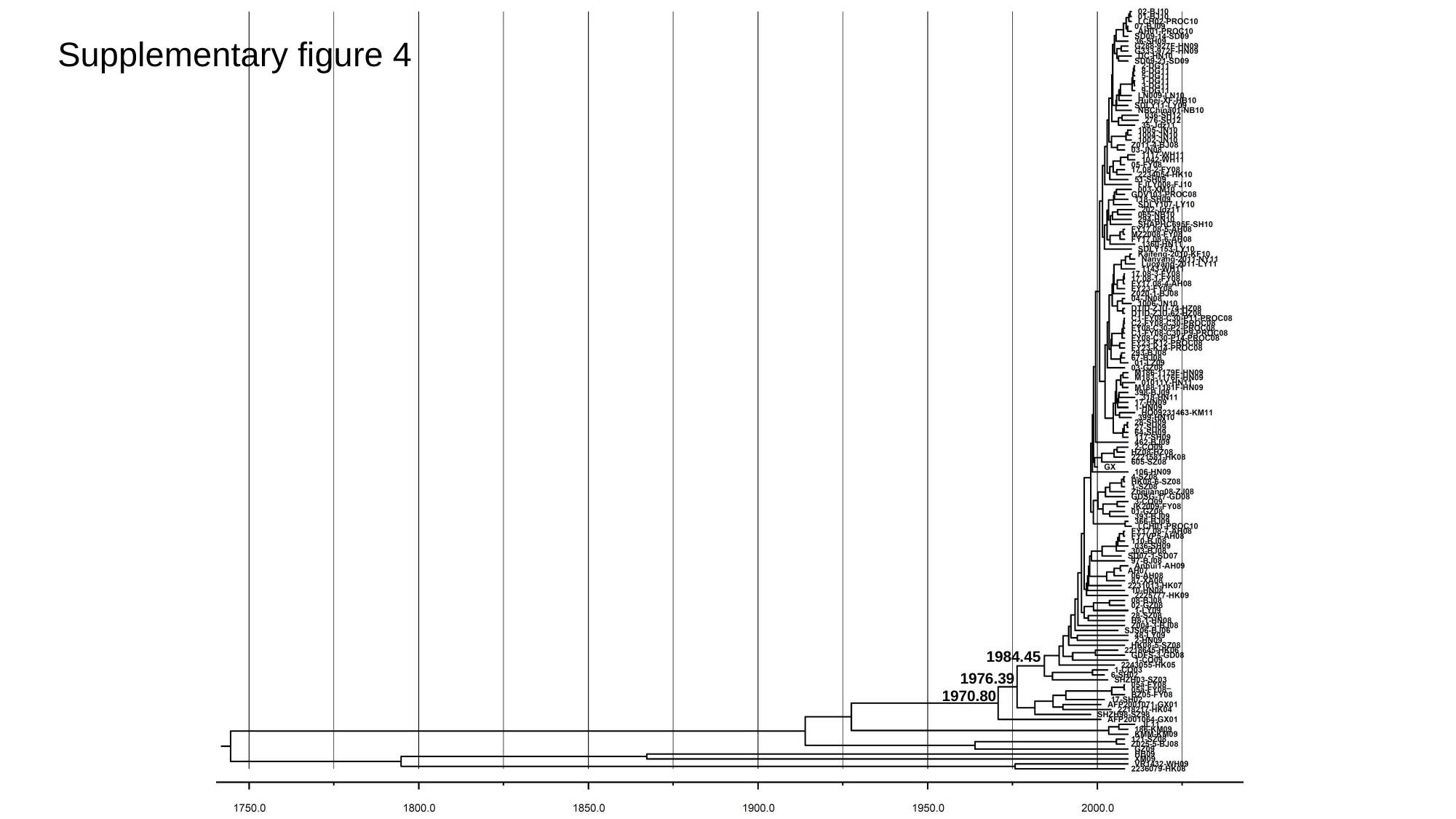

Supplementary figure 4
1984.45
1976.39
1970.80

Supplement: Additional file 5: Figure S4 — Bayesian MCMC analysis phylogeny of EV71 strains according to 3D coding region in China. A total of 154 complete 3D sequences of genotype C4 in China with known sampling dates were used to construct phylogeny as indicated. The tree was shown in a decreasing ordering, and the estimated dates of common ancestors of nodes were indicated at the nodes. [file 1423-0127-21-33-S5.pptx]
